# The influence of reflection and self-system on the effectiveness of self-regulation of students’ mental states

**DOI:** 10.1192/j.eurpsy.2022.1606

**Published:** 2022-09-01

**Authors:** M. Kartasheva, A. Prokhorov, A. Chernov, M. Yusupov

**Affiliations:** Kazan Federal University, Institute Of Psychology And Education, Kazan, Russian Federation

**Keywords:** self-regulation, academic activity, mental state, self-system

## Abstract

**Introduction:**

In the modern era of instability the problems of human adaptive abilities and mental regulation have become more and more relevant. The study of the processes of mental regulation is impossible without understanding the role of the mental structures: reflection and self-system of personality.

**Objectives:**

The purpose of the research is to study the interaction and mutual influence of the components of the self-system and reflective structures on the process of mental regulation of students.

**Methods:**

52 first year students were offered to complete questionnaires of reflectivity (M. Grant), of self-system (S. Pantileev), as well as the authors’ method of self-regulation effectiveness of mental states. Also we used Spearman’s rank correlation coefficient.

**Results:**

It is revealed the positive direction of the relationships between the reflection and the components of the self-system (p ≤ 0.05). Respondents with a high level of self-system are more successful in coping with the processes of mental regulation. The indicators of internal conflict lead to a decrease in the effectiveness of self-regulation (p ≤ 0.05). The ability of a person to evaluate himself and other people has a positive effect on the success of mental regulation (p ≤ 0.05). However, the excessive desire to reflect on past events is not conducive to self-regulation.

**Conclusions:**

The applied value of the research consists in the creation of a methodology that makes it possible to increase the regulatory and adaptive abilities of students during the educational process. Acknowledgements. This work was supported by the RFBR grant No. 20-013-00076.

**Disclosure:**

No significant relationships.

